# Early Assessment of Chemotherapy Response in Advanced Non-Small Cell Lung Cancer with Circulating Tumor DNA

**DOI:** 10.3390/cancers14102479

**Published:** 2022-05-18

**Authors:** Stephanie J. Yaung, Corinna Woestmann, Christine Ju, Xiaoju Max Ma, Sandeep Gattam, Yiyong Zhou, Liu Xi, Subrata Pal, Aarthi Balasubramanyam, Nalin Tikoo, Claus Peter Heussel, Michael Thomas, Mark Kriegsmann, Michael Meister, Marc A. Schneider, Felix J. Herth, Birgit Wehnl, Maximilian Diehn, Ash A. Alizadeh, John F. Palma, Thomas Muley

**Affiliations:** 1 Roche Sequencing Solutions, Inc., Pleasanton, CA 94588, USA; xiaoju.ma@gmail.com (X.M.M.); liu.xi@roche.com (L.X.); palma2112@yahoo.com (J.F.P.); 2 Roche Sequencing Solutions, Inc., 14473 Potsdam, Germany; corinna.woestmann@roche.com; 3 Roche Molecular Systems, Inc., Pleasanton, CA 94588, USA; shen.cju@gmail.com (C.J.); sandeep.gattam@contractors.roche.com (S.G.); yiyong.zhou@roche.com (Y.Z.); subrata.pal.sp2@roche.com (S.P.); b.aarthi@gmail.com (A.B.); 4 Alector, Inc., South San Francisco, CA 94080, USA; nalin.tikoo@alector.com; 5 Diagnostic and Interventional Radiology with Nuclear Medicine, Thoraxklinik, University Hospital, 69126 Heidelberg, Germany; hsl19@uni-heidelberg.de; 6 Diagnostic and Interventional Radiology, University Hospital, 69120 Heidelberg, Germany; 7 Translational Lung Research Centre (TLRC) Heidelberg, Member of the German Centre for Lung Research (DZL), 69120 Heidelberg, Germany; michael.thomas@med.uni-heidelberg.de (M.T.); michael.meister@med.uni-heidelberg.de (M.M.); marc.schneider@med.uni-heidelberg.de (M.A.S.); felix.herth@med.uni-heidelberg.de (F.J.H.); thomas.muley@med.uni-heidelberg.de (T.M.); 8 Translational Research Unit, Thoraxklinik at Heidelberg University Hospital, 69126 Heidelberg, Germany; 9 Institute of Pathology, University Hospital Heidelberg, 69120 Heidelberg, Germany; mark.kriegsmann@med.uni-heidelberg.de; 10 Roche Diagnostics GmbH, 82377 Penzberg, Germany; birgit.wehnl@roche.com; 11 Stanford Cancer Institute, Stanford University, Stanford, CA 94305, USA; diehn@stanford.edu (M.D.); arasha@stanford.edu (A.A.A.)

**Keywords:** ctDNA, NSCLC, chemotherapy, NGS, early molecular response

## Abstract

**Simple Summary:**

An early assessment of response to treatment is crucial to informing appropriate therapeutic management. Using a plasma-only strategy, we measured changes in circulating tumor DNA (ctDNA) levels after one or two cycles of chemotherapy in 92 patients with advanced non-small-cell lung cancer (NSCLC) treated with first-line chemo- or chemoradiation therapies. A ≤50% decrease in ctDNA level after one cycle of chemotherapy was associated with shorter progression-free survival and overall survival. A ≤50% decrease in ctDNA level after two cycles of chemotherapy also had shorter survival. Our findings demonstrate that using liquid biopsies to measure early changes in ctDNA levels in response to chemotherapy may help identify non-responders before standard-of-care imaging in advanced NSCLC. Monitoring treatment efficacy earlier and accurately can enable more personalized regimens to improve patient outcomes.

**Abstract:**

Monitoring treatment efficacy early during therapy could enable a change in treatment to improve patient outcomes. We report an early assessment of response to treatment in advanced NSCLC using a plasma-only strategy to measure changes in ctDNA levels after one cycle of chemotherapy. Plasma samples were collected from 92 patients with Stage IIIB-IV NSCLC treated with first-line chemo- or chemoradiation therapies in an observational, prospective study. Retrospective ctDNA analysis was performed using next-generation sequencing with a targeted 198-kb panel designed for lung cancer surveillance and monitoring. We assessed whether changes in ctDNA levels after one or two cycles of treatment were associated with clinical outcomes. Subjects with ≤50% decrease in ctDNA level after one cycle of chemotherapy had a lower 6-month progression-free survival rate (33% vs. 58%, HR 2.3, 95% CI 1.2 to 4.2, log-rank *p* = 0.009) and a lower 12-month overall survival rate (25% vs. 70%, HR 4.3, 95% CI 2.2 to 9.7, log-rank *p* < 0.001). Subjects with ≤50% decrease in ctDNA level after two cycles of chemotherapy also had shorter survival. Using non-invasive liquid biopsies to measure early changes in ctDNA levels in response to chemotherapy may help identify non-responders before standard-of-care imaging in advanced NSCLC.

## 1. Introduction

Lung cancer remains the leading cause of cancer-related mortality worldwide [1], with only 10–30% of patients surviving five years after diagnosis [2]. The latest analysis of epidemiological data on non-small cell lung cancer (NSCLC) in the United States suggests the introduction of more effective treatments and earlier treatment associated with earlier diagnosis may have increased the five-year survival estimate from 23.3% in 2014 to 26.4% in 2017 [3]. With the growing number of available treatment options, such as molecular targeted therapies and immunotherapies, monitoring response to therapy during initial cycles of treatment may enable an earlier change in treatment to further improve patient outcomes. However, treatment response in solid tumors is primarily based on radiological assessments, which are recommended from every 6 to 8 weeks or from 6 to 12 weeks for advanced NSCLC [4]. Performing serial computed tomography (CT) scans at short intervals from 3 to 4 weeks can detect changes in tumor size after one cycle of chemotherapy and potentially change clinical management [5], though standard methods such as Response Evaluation Criteria in Solid Tumors (RECIST) [6] are limited by practicability, radiation exposure, and challenges in morphological evaluation [7].

Liquid biopsies have emerged as a promising tool for precision oncology [8]. In 2018, the International Association for the Study of Lung Cancer (IASLC) issued a statement paper concluding that clinical implementation of liquid biopsy approaches is justified in several therapeutic settings relevant to NSCLC, particularly for initial molecular diagnosis when tumor tissue is unavailable and for sparing an invasive re-biopsy to determine subsequent therapy after progression on a targeted therapy [9]. Furthermore, quantifying changes in the levels of tumor-specific mutations in circulating tumor DNA (ctDNA) enables early response prediction and monitoring in various advanced solid tumors [10], including colorectal cancer [11,12,13] and breast cancer [14,15]. In advanced lung cancer, changes in ctDNA levels can be used as a biomarker to monitor early response to immunotherapy [16,17,18,19,20] or tyrosine kinase inhibitors (TKIs) [21,22,23,24].

Despite novel treatment advances, chemo- and chemoradiation therapy remain standard-of-care options in certain settings for treating advanced NSCLC [4]. A limited number of studies have explored the use of changes in ctDNA levels to monitor early response to chemotherapy. Previous work tracked changes in mutant levels of single genes in ctDNA, such as KRAS and TP53 [25,26,27], or examined ctDNA positivity rather than a change from baseline [28,29]. Moreover, analyses of ctDNA typically leverage prior knowledge of variants from matched tissue biopsies [29,30]. Since a tissue biopsy is often inaccessible in many late-stage lung cancer patients [31], we sought to determine whether variants found in baseline plasma ctDNA can also enable longitudinal monitoring without any prior knowledge of variants from matched tissue biopsies. Using a targeted next-generation sequencing (NGS) panel optimized for surveillance and monitoring of lung cancer, we hypothesized that a plasma-only approach measuring changes in ctDNA levels can assess early molecular response to chemo- or chemoradiation therapies in advanced NSCLC.

## 2. Materials and Methods

### 2.1. Study Design and Population

The Lung Cancer Multi-Marker Study was an observational, prospective clinical research study at Thoraxklinik, Heidelberg University Hospital, Germany. Eligible treatment-naïve subjects with histologically or cytologically proven late-stage adenocarcinoma or squamous cell carcinoma of the lung were enrolled in this study between December 2015 and March 2018 (Table 1). Blood samples from subjects were collected during regular clinical assessments at baseline, prior to the start of therapy, and throughout treatment. Response Evaluation Criteria in Solid Tumors guidelines (RECIST 1.1) [6] were followed when assessing the efficacy of treatment. The study was approved by the local ethics committee (S-611/2017) and health authority and carried out in accordance with Good Clinical Practice and the Declaration of Helsinki: Ethical Principles for Medical Research Involving Human Patients. Informed consent was obtained from all donors.

**Figure 1:**
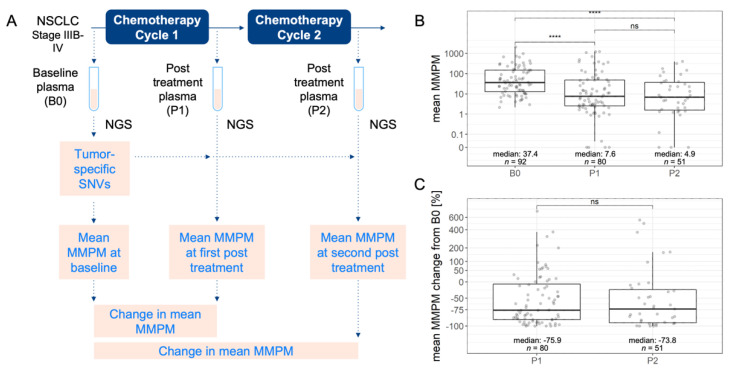
Study design and ctDNA level distributions. (**A**) ctDNA sequencing was performed on baseline plasma (B0) and first available plasma after one cycle of chemotherapy (P1) and first available plasma after two cycles of chemotherapy (P2). ctDNA levels were measured as mean mutant molecules per milliliter (MMPM). (**B**) Distribution of ctDNA levels in B0, P1, and P2 samples. (**C**) Distribution of changes in ctDNA level at P1 from B0 and P2 from B0 in matched samples. Center line indicates the median value. **** Wilcoxon *p* ≤ 0.0001. ns: not significant, Wilcoxon *p* > 0.05.

For our assessment of plasma-based measurements within the first one or two cycles of chemotherapy, we analyzed up to three plasma samples per subject (Figure 1A). The baseline (B0) plasma sample was the last sample collected prior to the treatment start date. The treatment start date was defined as the date of initiation of cycle 1 of chemotherapy in first line. The first post-treatment (P1) plasma sample was the first sample collected between 21 and 49 days after treatment started. The P1 sample had to be collected on or before the initiation of cycle 2 of chemotherapy in first-line, or on or before initiation of second-line therapy. The second post-treatment (P2) plasma sample was the first sample collected between 21 and 49 days after the start of chemotherapy cycle 2. The P2 sample had to be collected on or before the initiation of cycle 3 of chemotherapy in first-line or on or before initiation of second-line therapy. For all time points, samples were not included if the patient received any radiotherapy within 14 days prior to plasma collection.

### 2.2. ctDNA Sequencing and Bioinformatics Analysis

Cell-free DNA (cfDNA) was isolated from plasma samples and prepared for sequencing using the AVENIO ctDNA Surveillance Kit (Roche; for research use only; not for use in diagnostic procedures). The analytical quality of the extracted DNA was evaluated using a quantitative polymerase chain reaction (qPCR) method as described by Saelee et al. [32] to maximize cfDNA input into the hybrid capture-based NGS workflow, which was based on cancer personalized profiling by deep sequencing (CAPP-Seq) technology [33]. The workflow utilizes a 198-kb panel designed to cover recurrently mutated genomic loci in lung and colorectal cancers as well as genes in the U.S. National Comprehensive Cancer Network (NCCN) Guidelines. Analysis was carried out as described in the instructions of the kit. For the 223 samples from 92 patients used in this study, the median input mass was 25.7 ng (range 5–290 ng), median paired-end reads per sample were 54.9 million (range 31.9–164.9 million), median percentage of on-target reads was 69.0% (range 31.5–78.9%), and median unique molecular depth was 4747 (range 781–11,883).

NGS analysis was completed with the AVENIO Oncology Analysis Software [34] (Roche; for research use only; not for use in diagnostic procedures), which incorporates bioinformatics methods from CAPP-Seq technology and integrated digital error suppression [35] to remove random PCR errors and stereotypical errors from technical artifacts. Somatic calling was performed on the unfiltered list of single nucleotide variants (SNVs) from the software. The somatic calling approach used a previously described machine learning model [36] that takes into account the variation in variant allele fraction (AF) and information on common germline variants in public databases (Exome Aggregation Consortium [37] release 0.3.1, 1000 Genomes [38] phase3v5b, and dbSNP [39] build 144). The model was trained to remove germline variants based on the lower variation in AFs of germline variants than somatic variants in longitudinal plasma samples from the same subject. In addition to the tumor-specific SNVs, filtered insertion/deletions (indels) from the AVENIO Oncology Analysis Software were included in downstream analyses as tumor-specific variants for each subject.

We performed longitudinal ctDNA analysis and measured changes in mean mutant molecules per milliliter (MMPM) in post-treatment plasma compared to baseline by tracking the AF of the tumor-specific variants identified in the baseline plasma (Figure 1A). The MMPM for each variant was quantified as the variant AF multiplied by the extracted mass (ng) and 330, then divided by the plasma volume (mL). The factor of 330 is the estimated number of haploid genome equivalents per ng of DNA based on the molecular weight of the human genome. In ctDNA analyses not informed by baseline plasma information, we calculated a mean MMPM from all somatic variants identified in post-treatment plasma.

### 2.3. ctDNA Thresholds for Molecular Response

A pre-specified threshold of >50% vs. ≤50% decrease in ctDNA level from baseline was selected to classify molecular responders vs. molecular non-responders. Selection of the threshold was based on review of prior studies, which included ctDNA decreases of either >50% or >30% for immunotherapy response [16,18,19] or >98% or >90% for EGFR TKI response [22,23]. Zou et al. [20] used a threshold of >50% ctDNA decrease at 6 weeks after second-line chemotherapy or immunotherapy. 

We also explored the possibility of establishing a threshold based on a single post-treatment sample, not informed by a subject-specific baseline sample. This approach to molecular monitoring of response is well-established in the clinical management of chronic myeloid leukemia treated with TKIs, where molecular response is measured by the log decrease in BCR-ABL1 transcript level from a standardized baseline [40,41]. Instead of requiring analysis of the baseline sample, analysis of ctDNA levels in a single post-treatment plasma could provide an assessment of treatment response. For this method, the P1 samples in our study were randomly split into 60% training and 40% test sets with stratification by stage, smoking history, performance status, and radiotherapy status. The optimal threshold identified by maximally selected LogRank statistics [42] in the training set for classifying responders vs. non-responders was then applied to the test set.

### 2.4. Statistical Analysis 

Subject baseline characteristics were summarized using proportions for categorical variables and median and interquartile ranges (IQR) for continuous variables. Tumor response assessment on the first CT scan after the post-treatment plasma collection date was used to evaluate the association with mean MMPM change after one cycle of chemotherapy using Fisher’s exact test and calculation of the odds ratio and associated 95% confidence interval. 

Kaplan–Meier estimates and log-rank tests were used to evaluate classification of subjects as molecular responders vs. non-responders based on the developed algorithms using pre-defined or optimal thresholds. The differences in median progression-free survival (PFS) and overall survival (OS) between classification groups were estimated based on the Kaplan–Meier method. Six-month PFS was defined as time from treatment initiation to the date of first documented progressive disease (PD) in first line or death within 182 days after treatment initiation. Subjects who did not have a documented PD or remained alive at 182 days were censored at either 182 days or the date last known alive at time of data cut, whichever was earlier. Twelve-month OS was defined as time from treatment initiation to the date of death from any cause within 365 days after treatment initiation. Subjects who remained alive at 365 days were censored at either 365 days or the date last known alive at time of data cut, whichever was earlier. PFS rate at the six-month time point and OS rate at the twelve-month time point were estimated using Kaplan–Meier methodology.

Association between the classification groups and survival (PFS and OS) was assessed using both unadjusted and adjusted Cox proportional hazards models. Univariable Cox models were performed to assess the association between each potential adjustment variable (histology, age, sex, smoking history [smoker vs. ex-smoker vs. never smoked], UICC [III vs. IV], ECOG at baseline [0 vs. 1 or 2], and radiotherapy status) and survival. Variables with clinical relevance (*p*-value < 0.1) were included as multivariable adjustments. 

Data were analyzed using R 3.6.1 (The R Foundation for Statistical Computing Platform, Vienna, Austria). All tests were two-sided and considered statistically significant at *p*-value < 0.05 unless specified otherwise.

## 3. Results

### 3.1. Cohort Baseline Characteristics

A total of 223 plasma samples from 92 stage IIIB-IV NSCLC subjects were analyzed. In total, 80 subjects had a baseline (B0) and post-chemotherapy cycle 1 (P1) plasma available for analysis. In total, 51 subjects had a B0 and post-chemotherapy cycle 2 (P2) plasma available for analysis. Approximately 60% of subjects had lung adenocarcinoma, and 40% had lung squamous cell carcinoma (Table 1). Most subjects received first-line doublet chemotherapy with a platinum-based agent (carboplatin or cisplatin) in combination with vinorelbine or pemetrexed (Table S1).

### 3.2. Quantification of Circulating Tumor DNA in Plasma 

P1 plasma was collected on treatment days 21–41 (median: 23) after chemotherapy initialization, and P2 plasma was collected on days 42–91 (median: 45) of chemotherapy. All subjects had at least one tumor-specific SNV, insertion, or deletion detected in baseline plasma, with a median of 10 somatic mutations that were most frequently found in lung cancer genes, including TP53, KRAS, and NPAP1 (Table S2). Each variant was quantified with the MMPM metric, and the ctDNA level in a plasma sample was summarized as the mean MMPM across the tumor-specific variants in that sample. The median mean MMPM in plasma samples was 37.4 at baseline, 7.6 at P1, and 4.9 at P2 (Figure 1B). The distribution of changes in mean MMPM at P1 and P2 compared to baseline shows that most subjects experienced a decrease in ctDNA levels after one cycle of chemotherapy, and this decrease persisted after two cycles of chemotherapy (Figure 1C).

### 3.3. Association of Change in ctDNA Level with Clinical Outcome

**Figure 2:**
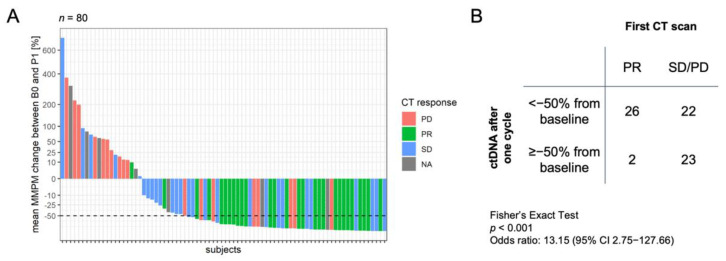
Association between first response assessment and ctDNA decrease after one chemotherapy cycle. (**A**) Waterfall plot showing change in ctDNA level at post-chemotherapy cycle 1 (P1) from baseline (B0). ctDNA levels were measured as mean mutant molecules per milliliter (MMPM). Each bar is colored by the first available response assessment after initiation of therapy: partial response (PR), stable disease (SD), progressive disease (PD), or CT scan not available (NA). Dotted line indicates the threshold of 50% decrease in mean MMPM. (**B**) Contingency table shows classification of response by ctDNA using a >50% decrease threshold vs. response by CT scan. *p*-value and odds ratio were calculated with Fisher’s Exact Test.

The tumor response assessment from the first available CT scan after treatment initiation (median of 20 days after P1 plasma collection) was compared with the change in ctDNA level between B0 and P1 plasma (Figure 2A). Most subjects who had a partial response had a decrease in ctDNA level. A >50% decrease (equivalent to <−50% change) in ctDNA level from baseline was significantly associated with partial response (OR 13.15, 95% CI 2.75–127.66, *p* < 0.001; Figure 2B). 

**Figure 3:**
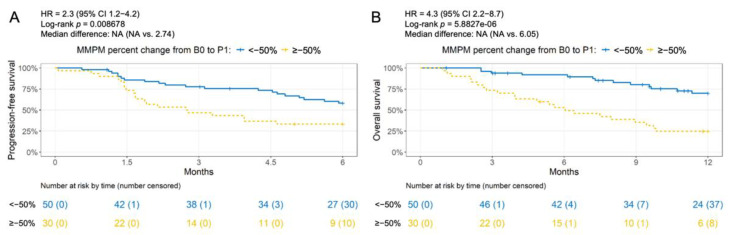
Association between survival and ctDNA decrease after one chemotherapy cycle. Progression-free survival (**A**) and overall survival (**B**) for >50% vs. ≤50% decrease in mean mutant molecules per milliliter (MMPM) from baseline in the first available plasma after one cycle of chemotherapy.

**Figure 4:**
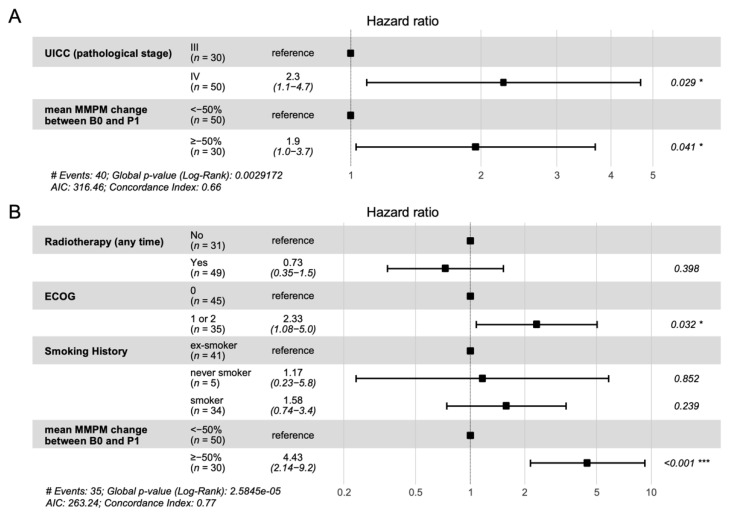
Multivariate Cox regression analysis of survival. Forest plots displaying hazard ratios from multivariate analysis of progression-free survival (**A**) and overall survival (**B**) for >50% vs. ≤50% decrease in mean mutant molecules per milliliter (MMPM) ctDNA. * *p* ≤ 0.05 *** *p* ≤ 0.001.

Subjects with ≤50% decrease in ctDNA level after one cycle of chemotherapy had a lower 6-month progression-free survival rate (Figure 3A; 33% vs. 58%, HR 2.3, 95% CI 1.2 to 4.2, log-rank *p* = 0.009) and a lower 12-month overall survival rate (Figure 3B; 25% vs. 70%, HR 4.3, 95% CI 2.2 to 8.7, log-rank *p* < 0.001). Unadjusted Cox proportional hazards models were performed for each potential adjustment variable to determine which ones to include in multivariate models determining associations between treatment response classification and PFS and OS (Table S3). A significant association between a ≤50% decrease in ctDNA level and PFS was seen after adjusting for stage (HR 1.9, 95% CI 1.0 to 3.7, log-rank *p* = 0.041; Figure 4A). Similarly, after adjusting for radiotherapy (received at any time during the study), ECOG performance status at baseline, and smoking history, a significant association was observed between a ≤50% decrease in ctDNA level and OS (HR 4.43, 95% CI 2.14 to 9.2, log-rank *p* < 0.001; Figure 4B). 

**Figure 5:**
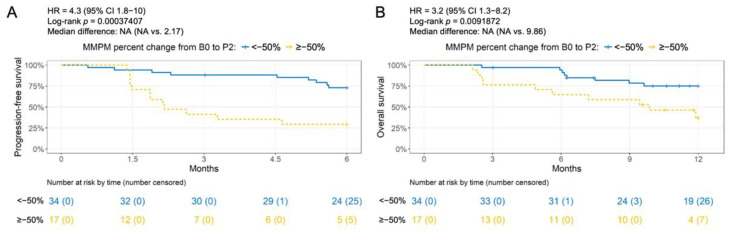
Association between survival and ctDNA decrease after two chemotherapy cycles. Progression-free survival (**A**) and overall survival (**B**) for >50% vs. ≤50% decrease in mean mutant molecules per milliliter (MMPM) from baseline in the first available plasma after two cycles of chemotherapy.

In the 51 subjects with both a baseline plasma and post-chemotherapy cycle 2 plasma, those with ≤50% decrease in ctDNA level in the P2 plasma had a lower 6-month progression-free survival rate (29% vs. 73%, HR 4.3, 95% CI 1.8 to 10, log-rank *p* < 0.001) and a lower 12-month overall survival rate (37% vs. 75%, HR 3.2, 95% CI 1.3 to 8.2, log-rank *p* = 0.009) (Figure 5).

### 3.4. Association of Single Time Point ctDNA Level with Clinical Outcome

The 80 subjects with a P1 plasma sample were randomly split into 60% training and 40% test sets with stratification by stage, smoking history, performance status, and radiotherapy status. For the stratification factors as well as other variables such as histology and age, there were no significant differences between the training and test sets (Figure S1A). By maximally selected LogRank statistics, an optimal threshold of 10 mean MMPM was identified for classifying OS in the 49 subjects in the training cohort. Subjects with a ≥10 mean MMPM had a worse 12-month OS (HR 4.7, 95% CI 1.7 to 13, log-rank *p* < 0.001) (Figure S1B). Using the same threshold for the test set of 31 subjects, those with ≥10 mean MMPM had worse 12-month OS (HR 4.6, 95% CI 0.99 to 21, log-rank *p* = 0.033) (Figure S1C). For PFS, an optimal threshold of 227 mean MMPM was identified. Subjects with ≥227 mean MMPM had worse 6-month PFS in the training cohort (HR 2.8, 95% CI 1.1 to 7.7, log-rank *p* = 0.031) and the test cohort (HR 5.5, 95% CI 1.6 to 19, log-rank *p* = 0.003) (Figure S2A). Given the distribution of mean MMPM values at P1 (Figure 1B), 227 is a very high cutoff that would classify only 14% of subjects as non-responders. Therefore, we also tried the threshold of 10 mean MMPM from the OS analysis to classify PFS. Although not statistically significant, subjects with ≥10 mean MMPM had worse 6-month PFS (Figure S2B).

There were not enough P2 samples (*n* = 51) for a train/test split, and, therefore, we did not analyze post-chemotherapy cycle 2 in single time point analyses.

## 4. Discussion

Our results suggest that ctDNA analysis using a targeted 197-gene NGS panel is a feasible approach for monitoring early treatment effect in subjects with advanced NSCLC treated with chemotherapy. We found that a ≤50% decrease in ctDNA levels after one or two cycles of chemotherapy was significantly associated with worse 6-month PFS and 12-month OS (Figure 3 and Figure 5). This finding is consistent with the results in Zou et al. [20], which used the same targeted NGS panel and reported that a ≤50% decrease from baseline in maximum AF at 6 weeks after second-line treatment initiation in the docetaxel cohort had worse OS, though the separation was not significant (*p* = 0.061). We observed a larger separation in the Kaplan–Meier curves in our study for a ≤50% decrease in ctDNA from baseline at the same time point (P2), possibly because the treatment effect could be more pronounced in a first-line setting. 

Another study utilizing a 22-gene NGS panel [28] found that the absence of ctDNA, not the decrease in ctDNA level, from 4 to 8 weeks after treatment initiation was the best prognostic marker; however, the analysis was based on a heterogeneous cohort of patients treated with either EGFR TKI or chemotherapy, precluding a direct comparison of chemotherapy response assessment. The smaller gene panel may limit the ability to detect tumor burden in plasma. In fact, this may explain the smaller decreases in ctDNA levels observed in single-gene studies, such as Jiang et al. [27], where patients with partial responses averaged a 31% drop in TP53 ctDNA levels after two cycles of chemotherapy. 

Patient coverage is also an important consideration; studies tracking KRAS in patients with advanced NSCLC could detect KRAS mutations in only 10% or 48% of baseline plasma [25,26]. In our study, 100% of the 92 subjects had at least one trackable baseline mutation with the 197-gene NGS panel. For subjects treated with targeted therapies, one can take advantage of the mutations in genes that are relevant to the corresponding targeted therapies for response monitoring [43]. For tumors with no known driver genes, there are no readily available tools to monitor the disease burden of these subjects due largely in part to the molecular heterogeneity of the disease. Earlier work described a way to monitor these subjects, but customized assays are necessary [44]. The current work demonstrated the feasibility of an NGS panel approach to achieve high mutation detection rates for lung cancers, which is consistent with other work that utilized CAPP-Seq technology [33,45].

Importantly, we relied on baseline plasma instead of tissue biopsy at diagnosis to identify tumor-specific variants to enable post-treatment monitoring. Previous studies on longitudinal monitoring have mainly used baseline tissue biopsies as a starting point to identify tumor-derived variants [29,33,44]. Since tissue is not always accessible in late-stage lung cancer subjects, a complementary approach is needed in the absence of a tissue biopsy. In this study, we demonstrate the feasibility of identifying tumor-specific variants in baseline plasma ctDNA that could enable longitudinal monitoring. Consistent with prior studies [33,45], most of the somatic variants we identified in the plasma of lung cancer subjects were in non-driver genes. However, the presence and level of these non-driver mutations correlated with disease burden and were useful for post-treatment response assessment. 

If baseline plasma is unavailable, a single-time point analysis of ctDNA level in post-treatment plasma could monitor therapy response. We identified a potential threshold of 10 mean MMPM in plasma after one cycle of chemotherapy to classify responders vs. non-responders. The same threshold was not significantly associated with 6-month PFS, suggesting that predicting PFS but not OS after treatment initiation requires quantification relative to a subject-specific baseline. This is consistent with the fact that progressive disease per RECIST criteria is measured by comparing tumor size from a baseline CT scan. Longitudinal measurement relative to baseline has been explored with other methods as well, such as a >20% reduction in standardized uptake volume by positron emission tomography after one cycle of chemotherapy [46], a >27% drop in serum levels of cytokeratin 19 fragment (CYFRA 21-1) after one cycle of chemotherapy [47], or a >20% decrease in serum levels of both carcinoembryonic antigen (CEA) and CYFRA 21-1 after two cycles of chemotherapy [48]. Besides measuring changes in ctDNA levels in plasma, other approaches could be considered for a multimodal approach for predicting progression. 

## 5. Conclusions

We found that quantification of changes in ctDNA level using a targeted NGS panel is a promising biomarker reflecting tumor dynamics and could identify subjects who had a higher risk of failing therapy one treatment cycle earlier than routine CT imaging. Moreover, an early assessment of the treatment effect can potentially be measured in plasma within the first treatment cycle. Our results demonstrate that ctDNA quantitation is potentially feasible for early assessment of the treatment response of solid tumors, especially when tissue is not available. We present a framework whereby longitudinal ctDNA analysis could enable real-time, non-invasive monitoring of cancer, providing novel insights into whether an early response assessed by changes in the mutant molecule level has the potential to predict treatment effect in a tissue-independent fashion. The cutoffs and parameters described in this study may be suitable for different clinical research settings depending on sample availability relative to treatment.

## 6. Patents

A.B., C.J., X.M.M., T.M., F.J.H., N.T., and B.W. have filed a patent (WO2019211418) on utilizing mutant molecule count for quantifying ctDNA.

## Figures and Tables

**Table 1:** Baseline characteristics of study subjects.

Characteristic	Subjects (*n* = 92)
Histology Subtype, *n* (%)	
Adenocarcinoma	56 (61)
Squamous cell carcinoma	36 (39)
Age, median (IQR)	65 (59, 71)
Sex, *n* (%)	
Female	23 (25)
Male	69 (75)
Smoking History, *n* (%)	
Ex-smoker	48 (52)
Never smoked	5 (5)
Smoker	39 (42)
UICC, *n* (%)	
IIIB	29 (32)
IIIC	9 (10)
IV	34 (37)
IVA	14 (15)
IVB	6 (7)
ECOG, *n* (%)	
0	55 (60)
1	36 (39)
2	1 (1)
Tumor Stage, *n* (%)	
T1	3 (3)
T2	15 (16)
T3	31 (34)
T4	42 (46)
TX	1 (1)
N Stage, *n* (%)	
N0	4 (4)
N1	6 (7)
N2	35 (38)
N3	46 (50)
NX	1 (1)
M Stage, *n* (%)	
M0	34 (37)
M1	54 (59)
MX	4 (4)

Note: IQR, interquartile range; UICC, Union for International Cancer Control; ECOG—0 = Fully active, able to carry on all pre-disease performance without restriction, 1 = Restricted in physically strenuous activity but ambulatory and able to carry out work of a light or sedentary nature, 2 = Ambulatory and capable of all self-care but unable to carry out any work activities; up and about more than 50% of waking hours.

## Data Availability

The data analyzed are available from the corresponding author on reasonable request.

## Supplementary Materials

The following supporting information can be downloaded at: https://www.mdpi.com/article/10.3390/cancers14102479/s1, Figure S1: Association between overall survival and ctDNA level after one chemotherapy cycle, Figure S2: Association between progression-free survival and ctDNA level after one chemotherapy cycle; Table S1: Summary of First Line Treatment Description, Table S2: Most Frequently Mutated Genes in Baseline Plasma (*n* = 92), Table S3: Hazard Ratios and Confidence Intervals for Potential Adjustment Variables.
