# Supplementary material for: Early Assessment of Chemotherapy Response in Advanced Non-Small Cell Lung Cancer with Circulating Tumor DNA
Source: Cancers (Basel). 2022 May 18;14(10):2479. doi: 10.3390/cancers14102479 (PMC9139958; doi:10.3390/cancers14102479)
Supplement: Supplementary file 1 [file cancers-14-02479-s001.zip › cancers-1716883-supplementary.pdf]

Supplementary Materials: Early Assessment of Chemotherapy Response in Advanced Non-Small Cell Lung Cancer with Circulating Tumor DNA

Stephanie J. Yaung, Corinna Woestmann, Christine Ju, Xiaoju Max Ma, Sandeep Gattam, Yiyong Zhou, Liu Xi, Subrata Pal, Aarthi Balasubramanyam, Nalin Tikoo, Claus Peter Heussel, Michael Thomas, Mark Kriegsmann, Michael Meister, Marc A. Schneider, Felix J. Herth, Birgit Wehnl, Maximilian Diehn, Ash A. Alizadeh, John F. Palma and Thomas Muley

A

Characteristic	Level	Test (n=31)	Train (n=49)	*p**
Histology Subtype (%)	adenocarcinoma	22 (71.0)	27 (55.1)	0.237
	squamous cell carcinoma	9 (29.0)	22 (44.9)	
UICC (%)**	III	11 (35.5)	19 (38.8)	0.953
	IV	20 (64.5)	30 (61.2)	
Sex (%)	Female	9 (29.0)	10 (20.4)	0.54
	Male	22 (71.0)	39 (79.6)	
Radiotherapy (%)**	not received	11 (35.5)	20 (40.8)	0.809
	received at any time	20 (64.5)	29 (59.2)	
ECOG (%)**	0	17 (54.8)	28 (57.1)	1
	1 or 2	14 (45.2)	21 (42.9)	
Smoking History (%)**	ex-smoker	15 (48.4)	26 (53.1)	0.933
	never smoked	2 (6.5)	3 (6.1)	
	smoker	14 (45.2)	20 (40.8)	
Age (median [IQR])		66.00 [59.50, 70.50]	66.00 [60.00, 73.00]	0.55

* Training and testing cohort were compared using Pearson's chi square, Fisher's exact test, or Wilcoxon rank-sum test as appropriate.
** Used as stratification factors.

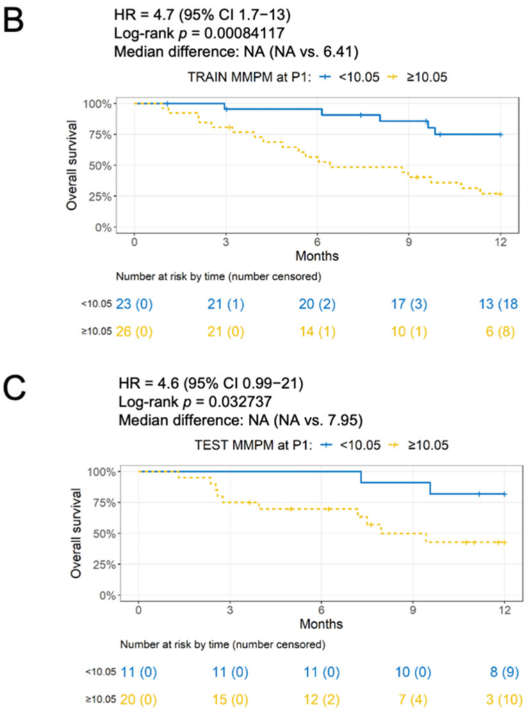

Figure S1. Association between overall survival and ctDNA level after one chemotherapy cycle. (**A**) Comparison of baseline characteristics between test and training cohorts. Overall survival for >10 vs. ≤10 mean mutant molecules per milliliter (MMPM) in the first available plasma after one cycle of chemotherapy in the training (**B**) and test (**C**) sets.

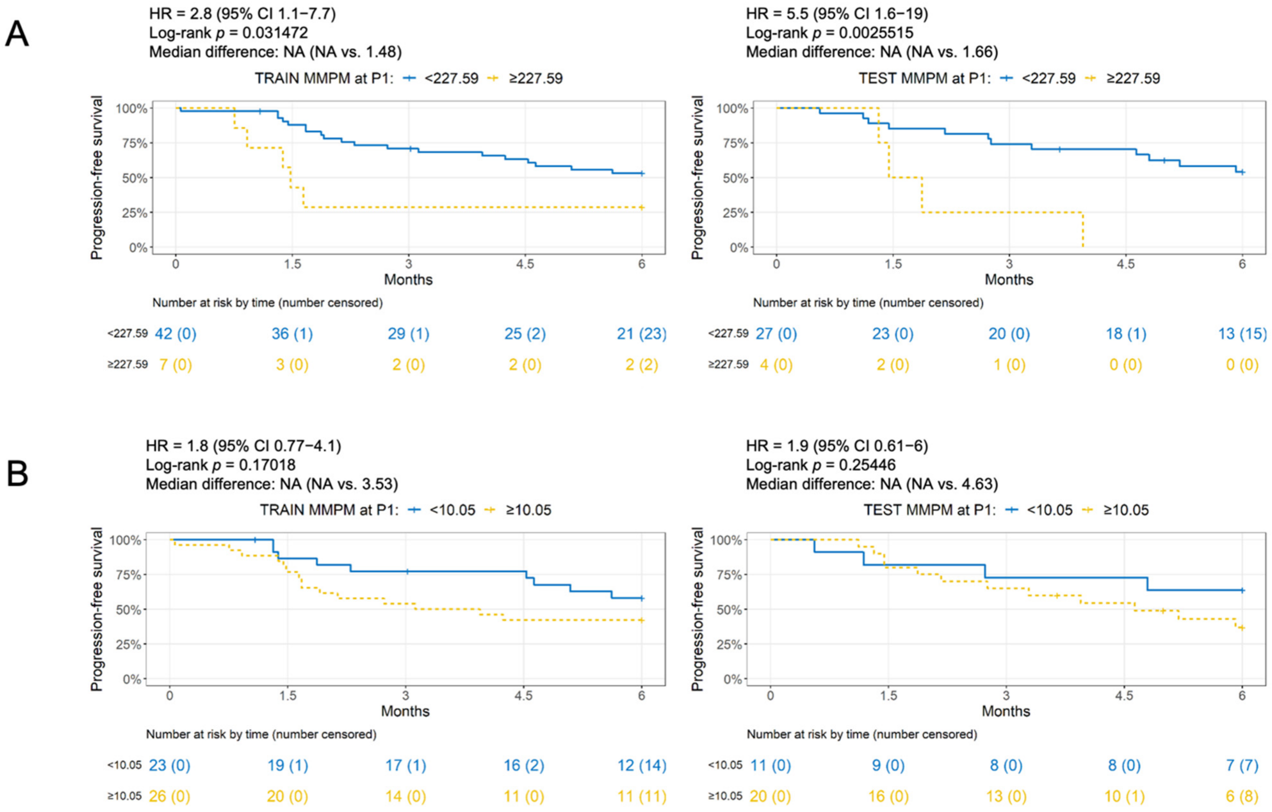

Figure S2. Association between progression-free survival and ctDNA level after one chemotherapy cycle. (**A**) Progression-free survival for >227 vs. ≤227 mean mutant molecules per milliliter (MMPM) in the first available plasma after one cycle of chemotherapy in the training (**left**) and test (**right**) cohorts. (**B**) Progression-free survival for >10 vs. ≤10 mean mutant molecules per milliliter (MMPM) in the first available plasma after one cycle of chemotherapy in the training (**left**) and test (**right**) cohorts.

Table S1. Summary of First Line Treatment Description.

First Line Treatment	Overall *n* (%)
Chemotherapy	92 (100)
Carboplatin or Cisplatin	91 (99)
Platinum–based Vinorelbine	49 (53)
Platinum–based Pemetrexed	27 (29)
Platinum–based Gemcitabine	6 (7)
Platinum–based Paclitaxel	3 (3)
Platinum–based Abraxane	2 (2)
Platinum–based Paclitaxel and Abraxane	2 (2)
Platinum–based Vinorelbine and Pemetrexed	1 (1)
Platinum–based Vinorelbine and Vincristine	1 (1)
Platinum–free Pemetrexed	1 (1)

Table S2. Most Frequently Mutated Genes in Baseline Plasma (*N* = 92).

Gene	*N* (%)
TP53	52 (57)
NPAP1	29 (32)
KRAS	27 (29)
EGFR	18 (20)
KIT	17 (18)
APC	16 (17)
BRCA2	16 (17)
FAM135B	16 (17)
POM121L12	15 (16)
SLC8A1	15 (16)
USP29	15 (16)
CSMD3	14 (15)
FBXL7	14 (15)
CHRM2	13 (14)
KIAA1211	13 (14)
LRRC7	13 (14)
MET	13 (14)
ALK	12 (13)
BRCA1	12 (13)
ERBB2	12 (13)
KEAP1	12 (13)
LRRTM1	12 (13)
LRRTM4	12 (13)
BRINP2	11 (12)
DDI1	11 (12)
KPRP	11 (12)
SLITRK5	11 (12)
ZNF521	11 (12)
BCHE	10 (11)
BRAF	10 (11)
BRINP3	10 (11)
MKRN3	10 (11)
NLRP3	10 (11)
PDGFRA	10 (11)
RET	10 (11)
SLITRK4	10 (11)

Note: Genes are only counted once per subject regardless of specific mutation. Mutations include both synonymous and nonsynonymous single nucleotide variants, insertions and deletions.

Table S3. Hazard Ratios and Confidence Intervals for Potential Adjustment Variables.

**Analysis for Full Population (*n* = 92)**	**PFS**		**OS**	
Variable	HR (95% CI)	logrank *p**	HR (95% CI)	logrank *p**
**Histology Subtype**		0.17		0.24
Squamous cell carcinoma	0.65 (0.35–1.20)		0.67 (0.34–1.32)	
Adenocarcinoma	Reference		Reference	
**Age**	1.00 (0.97–1.03)	0.91	1.02 (0.98–1.06)	0.33
**Sex**		0.14		0.30
Male	0.62 (0.33–1.17)		0.70 (0.35–1.38)	
Female	Reference		Reference	
**Smoking History**		0.58		**0.090**
Never Smoked	1.41 (0.33–6.00)		1.99 (0.45–8.74)	
Smoker	1.36 (0.75–2.47)		2.05 (1.05–3.98)	

Ex-Smoker	Reference		Reference	
**ECOG**		0.71		**0.0053**
1 or 2	1.12 (0.62–2.04)		2.43(1.28–4.62)	
0	Reference		Reference	
**UICC**		**0.0061**		0.14
IV	2.40 (1.26–4.59)		1.67 (0.84–3.32)	
III	Reference		Reference	
**Radiotherapy (any time)**		0.28		**0.088**
Received	0.72 (0.40–1.30)		0.58 (0.30–1.09)	
Not received	Reference		Reference	
**Radiotherapy (within 365 days)**		0.39		0.14
Received	0.77 (0.43–1.39)		0.62 (0.33–1.17)	
Not received	Reference		Reference	
**Radiotherapy (within 182 days)**		0.28		0.41
Received	0.73 (0.40–1.30)		0.76 (0.40–1.45)	
Not received	Reference		Reference	

Note: Abbreviations: PFS, progression free survival; OS, overall survival. *Global *p*-values presented for categorical variables with more than 2 levels. Unadjusted Cox proportional hazards models were performed for each potential adjustment variable. *p*-values in bold are below the 0.1 threshold used to select variables for inclusion in multivariate Cox proportional hazards models.
